# BioRssay: an R package for analyses of bioassays and probit graphs

**DOI:** 10.1186/s13071-021-05146-x

**Published:** 2022-01-24

**Authors:** Piyal Karunarathne, Nicolas Pocquet, Pierrick Labbé, Pascal Milesi

**Affiliations:** 1 Department of Ecology and Genetics, Evolutionary Biology Centre, Uppsala University, Norbyvägen 18D, SE-752 36, Uppsala, Sweden; 2 Institut Pasteur de Nouvelle-Calédonie, URE-Entomologie Médicale, Nouméa, New Caledonia; 3 Institut Universitaire de France, 1 Rue Descartes, 75231 Cedex 05 Paris, France; 4 Institut Des Sciences de L’Evolution de Montpellier (UMR 5554, CNRS-UM-IRD-EPHE), Université de Montpellier, 34095 Cedex 5 Montpellier, France; 5 SciLifelab, Uppsala, Sweden

**Keywords:** Bioassays, Probit analysis, Dose–response, Exposure–response, Lethal dose, Lethal exposure

## Abstract

Dose–response relationships reflect the effects of a substance on organisms, and are widely used in broad research areas, from medicine and physiology, to vector control and pest management in agronomy. Furthermore, reporting on the response of organisms to stressors is an essential component of many public policies (e.g. public health, environment), and assessment of xenobiotic responses is an integral part of World Health Organization recommendations. Building upon an R script that we previously made available, and considering its popularity, we have now developed a software package in the R environment, *BioRssay*, to efficiently analyze dose–response relationships. It has more user-friendly functions and more flexibility, and proposes an easy interpretation of the results. The functions in the *BioRssay* package are built on robust statistical analyses to compare the dose/exposure–response of various bioassays and effectively visualize them in probit-graphs.

**Graphical Abstract:**

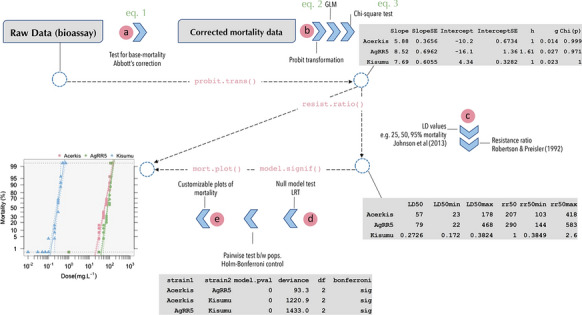

## Background

Bioassays aim at evaluating the potency of a compound. They usually consist in measuring the response of a “population” (e.g. organisms, populations, tissues, cells lines, strains, etc.) to increasing doses (or intensities or exposure times) of a stimulus (most of the time a xenobiotic or a chemical) to quantify specific dose–response relationships (also known as exposure–response relationships). Because of their effectiveness, dose–response relationship analyses are widely used in a large spectrum of scientific disciplines (e.g. epidemiology, microbiology, toxicology, environment quality monitoring, vector and pest control, and parasite biology). A prime example of such analyses is the monitoring of resistance to xenobiotics. Since the 1950s, xenobiotics (e.g. insecticides, pesticides, fungicides) have been widely used to control populations of vectors or pests [1]. However, as a counter-result, resistance mechanisms to such substances have been selected in targeted populations, undermining their efficiency [2]. Therefore, establishing and comparing the resistance levels of various populations to various xenobiotics is at the core of the World Health Organization (WHO) recommendations in order to define/adjust vector control strategies. These resistance assessments are usually done by exposing batches of individuals (adults or larvae) to varying doses of the xenobiotic to assess their responses (e.g. mortality or knockdown effect).

Despite bioassays being used in many fields, there has been a substantial lack of easily accessible statistical infrastructure for their analysis. In 2013, we developed an R script with a robust statistical background to describe and compare dose–mortality relationships [3], and it has since been used in several similar studies (e.g. [4–10]). In order to make it more user-friendly and easily accessible to the scientific community, we have now developed it into an R package called *BioRssay*, with more flexibility and an improved presentation of the results.

## Workflow

**Fig. 1:**
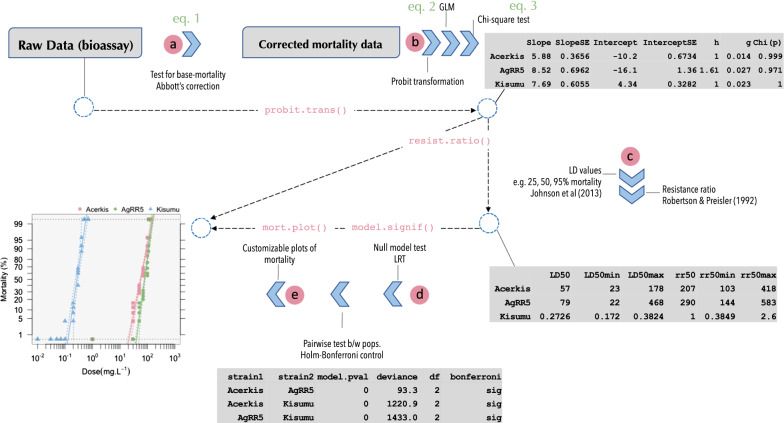
General workflow of the BioRssay package. Solid blue arrows represent different steps in the workflow; dashed arrows are associated with the function used in the *BioRssay* R package to execute these steps. The letters refer to the descriptions in the main text. Screenshots of software output are included

*BioRssay* is a comprehensive compilation of scripts in R language [11] designed to analyze dose–response relationships (or exposure–response: mortality, knockdown effect, etc.) from bioassays of one or more strains, lines, tissues, cells, etc. [hereafter referred to as “population(s)”]. This package provides a complete analytic workflow, from data quality assessment to statistical analyses and data visualization, as follows (see also Fig. 1).

**Fig. 2:**
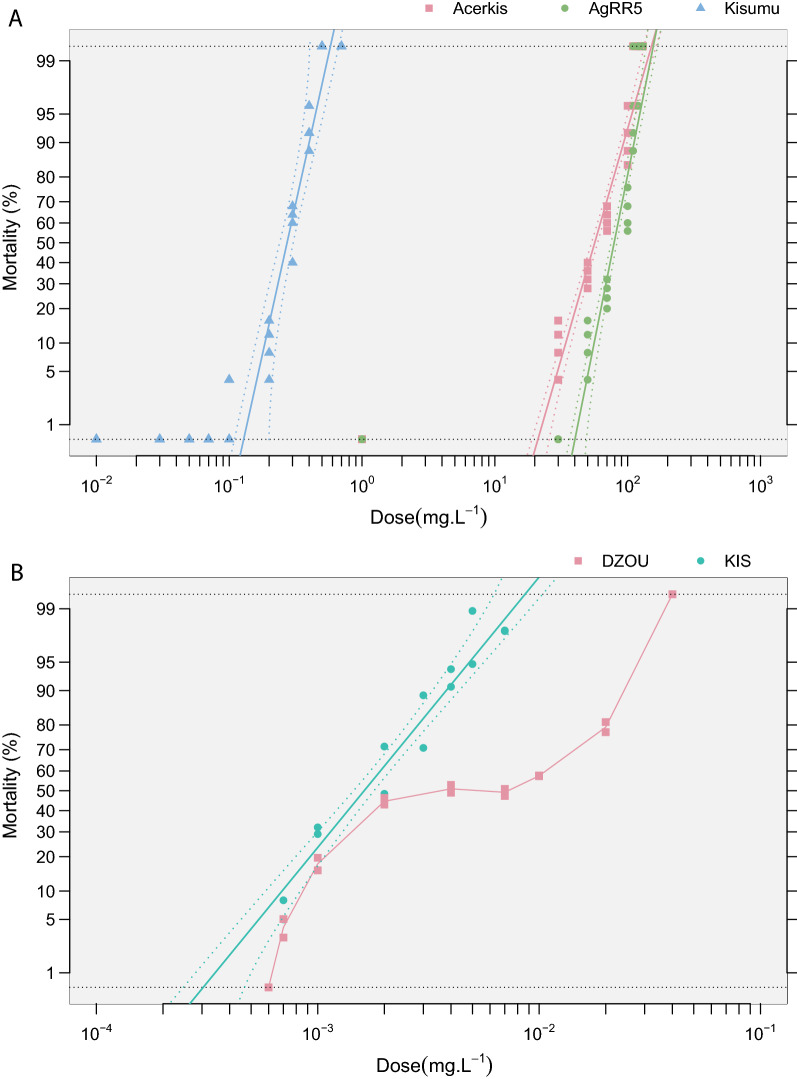
Probit graphs generated by the BioRssays. **a** Linear relationships between probit-transformed mortality rates and log-dose of bendiocarb insecticide for different mosquito populations (data from [3]). Kisumu (blue triangles) is the susceptible reference strain. AcerKis (red square) and AgRR5 (green circles) show resistance levels significantly higher than that of the reference population, AgRR5 showing the strongest resistance level (Fig. 1 and Table 2). **b** Same as **a** but for KIS (green dots) and DZOU (red squares) populations exposed to temephos insecticide. Note that the relation is not linear for the DZOU population, and dots are connected by segments (unpublished data)

- In the first step, the base mortality in the controls (i.e. a sample of the population not exposed to the tested stimulus) is taken into account to control for the mortality (or effect) associated with the experiment itself, regardless of the exposure (this can be critical when the exposure requires a long period). Data are adjusted following Abbott's formula using a maximum likelihood approach (*optim* function, method “L-BFGS-B”, *stats* R package [11]), to estimate correction factors (Eq. 1), while taking into account heterogeneity in the mortality rates between replicates (see example in Table 1; NB: users should be cautious in their use of the data if the heterogeneity is high) [12]:
  1 $$1- \frac{{m}_{T}}{{m}_{C}},$$
  where *m*_T_ and *m*_C_ are the survival rates in the treatments and the controls, respectively. By default, the correction is applied if mortality in the control is higher than 5%, but any threshold can be specified.
- Then, for each population independently, a generalized linear model (GLM) is fitted to the probit-transformed mortality rates, with the quasi-binomial family to account for possible overdispersion (Eq. 2); if Abbott’s correction has been applied, the model is fitted to the adjusted mortality rates (see above, point a).
  2 $$E\left({\Phi }^{-1}(P(Y=1|X)\right)=\beta X,$$
  where ${\Phi }^{-1}$ is the probit function, *Y* is the mortality rates, *X* is a design matrix, and $\beta$ the vector of effects of the covariables included in the design matrix (intercept and dose). The estimated effects (i.e. slope and intercept, with their standard errors) are reported. The slope and the intercept are used to test the linearity of the log-dose response using a Chi-square test of homogeneity (Eq. 2) between the model predictions and the probit-transformed data; significant deviations from linearity may for example reflect mixed populations, or a threshold effect.
  3 $${\chi }^{2}=\sum \frac{{\left(\mathrm{observed}-\mathrm{predicted}\right)}^{2}}{\mathrm{predicted}},$$
- The parameters estimated using Eq. 2 are then used to compute lethal doses (LD, also lethal concentrations, LC) with their corresponding 95% confidence intervals (CI), following the approach developed by Johnson et al. [13] from Finney [14], which allows us to take the heterogeneity of the data (*h*) into account, with larger *h* leading to larger CI. Heterogeneity-related parameters (*h* and *g* in Table 2) are reported; according to Finney's recommendation: “With almost all good sets of data, *g* will be substantially smaller than 1.0 and seldom greater than 0.4.” By default, LD are computed at 25%, 50%, and 95% of mortality; alternatively, any level of LD and CI can be specified by the user. Resistance ratios (RR), with their 95% CI, are calculated according to Robertson and Preisler [15]. They measure the magnitude of the difference in dose–responses of two populations when exposed to the same stimulus. For a given LD, the LD of a focal population is divided by the LD of the population with the lowest one (usually a susceptible reference, Table 2).
- When several populations (or strains, lines, etc.) are exposed to the same stimulus, we offer the possibility to test whether they present significant differences in their responses (slope and/or magnitude). We use a likelihood ratio test (LRT) to compare a null model, assuming that all the data come from the same population (i.e. where the dose is the only covariable in the design matrix, *X* in Eq. 2), with the complete model where the dose, the population effect, and their interaction are included in the design matrix *X* (Eq. 2). As in point b, the effects are fitted with the *glm* function in R, with the quasi-binomial family to account for overdispersion.
  With only two populations (e.g. comparison between an unknown population and a reference), a significant difference between the two models indicates that these populations differ in the magnitude of the response and/or slope. With more than two populations, it means that *at least* two populations show differing responses in the magnitude of the response and/or slope. In this case, pairwise comparisons (post hoc LRT) are implemented to identify the differing populations, testing for statistical significance of the difference between all population pairs; the Holm–Bonferroni method is then used to control for the family-wise error inherent in multiple testing [16].
  Note that only populations that pass the linearity test should be compared (see above point b): If at least one of the populations failed the linearity test, the validity of comparing the slope or magnitude of their dose-mortality response is, at best, highly questionable (not recommended). These populations should be presented, but only as such (accordingly, no regression is fitted to these populations by *BioRssay*, see point e). Nonetheless, it is still valid to compare the slope and magnitude between the remaining populations, i.e. those that show a linear log-dose response.
- Customizable (confidence levels, colors, symbols, etc.) plots of the probit-transformed regressions are drawn in the last step (Fig. 2a, b). By default, if the probit-transformed response significantly deviates from linear regression, the data are connected by segments; the (invalid) regression and associated CI are not plotted (e.g. Fig. 2b, DZOU population, red squares). Confidence intervals around the regressions can be removed or added, and users can specify any levels of CI.

**Table 1:** A subset of bioassays conducted on three strains of *Anopheles gambiae* mosquitoes exposed to increasing doses of temephos insecticide (data from [5])

Insecticide	Strain	Dose	Total	Dead	Replicate	Date	Mort	Probmort
Temephos	KIS-ref	0.002	97	47	1	2011-01-26	0.481	−0.045
Temephos	KIS-ref	0.003	96	68	1	2011-01-26	0.706	0.544
Temephos	KIS-ref	0.004	98	89	1	2011-01-26	0.907	1.326
Temephos	DZOU	0.001	97	4	1	2010-08-04	0.041	−1.731
Temephos	DZOU	0.002	97	20	1	2010-08-04	0.208	−0.812
Temephos	DZOU	0.004	100	31	1	2010-08-04	0.312	−0.487
Temephos	DZOU	0.007	95	52	1	2010-08-04	0.542	0.107
Temephos	DZOU2	0.002	97	24	1	2010-08-04	0.250	−0.674
Temephos	DZOU2	0.004	100	35	1	2010-08-04	0.353	−0.376
Temephos	DZOU2	0.007	95	56	1	2010-08-04	0.585	0.215
Temephos	DZOU2	0.010	97	69	1	2010-08-04	0.708	0.5485

The four columns “Strain”, “Dose”, “Total”, “Dead” are the mandatory input format. *Mort* and *Probmort* are Abbott’s corrected mortalities and probit-transformed mortalities, respectively

**Table 2:** Parameters estimated from the probit-transformed data

Populations	Linear regression parameters					LD_50_			RR_50_		
	Slope (± SE)	Intercept (± SE)	Chi(*p*)^a^	*h*	*g*	LD	LD_min_	LD_max_	RR	RR_min_	RR_max_
AcerKis^b^	5.88 ± 0.37	−10.30 ± 0.67	1.00	1.00	0.02	57	23	178	207	103	418
AgRR5^b^	8.52 ± 0.70	−16.17 ± 1.36	0.97	1.61	0.03	79	22	468	290	144	583
Kisumu^b^	7.69 ± 0.61	4.34 ± 0.33	1.00	1.00	0.02	0.27	0.17	0.38	–	–	–
DZOU^c^	1.64 ± 0.19	3.77 ± 0.45	< 1e−3	7.94	0.06	5e−3	2e−4	0.041	3.07	2.77	3.4
KIS^c^	3.46 ± 0.26	9.64 ± 0.72	0.36	3.09	0.03	2e−3	1e−4	0.01	–	–	–

^a^Chi(*p*) is the *p*-value of the Chi-square test of homogeneity

^b^AcerKis, AgRR5, and Kisumu are different populations of *An. gambiae* exposed to bendiocarb, Fig. 2a (data from [3])

^c^DZOU and KIS are different populations of *An. gambiae* exposed to temephos, Fig. 2b (unpublished data)

For each insecticide, Kisumu and KIS populations respectively have the lowest LD_50_ and were used as the references to compute the respective resistance ratios (RR_50_)

## Benchmarking BioRssay

What makes *BioRssay* stand out for dose–response analyses and how can it complement other similar R packages and functions? We discuss three R packages that provide dose–response analysis: *drc* [17], *protti* [18] and *lava* [19].

- The core of the *drc* package is to provide the user with a comprehensive set of model fitting followed by dose–response analysis [17], while *BioRssay* has been designed primarily to facilitate the analysis of bioassay data in a ready-to-go approach. As such, *BioRssay* is optimized to compare dose–response between many different populations exposed to the same stimulus. Relevant quantities, as LD and RR, along with their CI, are automatically generated, and a function (*model.signif*) is dedicated to the comparison of dose–responses. Producing these results would require combining the output of several functions from *drc* packages (e.g. *drm*, *ED*, *ED.comp*, *comped*, *comParm*, *predict.drc, anova.drc*). Another main difference stems from the use of a quasi-binomial for model fitting in *BioRssay* to account for possible data overdispersion, which is not possible in *drc,* despite the variety of models implemented. Finally, *BioRssay* also includes data preprocessing by implementing Abbott’s correction for mortality in controls before fitting the dose–response. The *drm* function in *drc* is much more thorough in terms of error model selection, allowing a dose–response analysis using for example binomial, Poisson, four- and five-parameter log-logistic models, and Weibull models. The *mselect* function of *drc* is handy in identifying the correct dose–response model for populations that fail the linearity test.
- The function *fit_drc_4p* from the package *protti* [18] is a wrapper function for the *drc* package’s *drm* function implementing a four-parameter log-logistic model.
- The function *PD* in the *lava* package [19] allows for dose–response calculation for binomial regression models.

## Package accessibility and concluding remarks

The package is available freely on GitHub at https://github.com/milesilab/BioRssay (https://doi.org/10.5281/zenodo.5172072). We also provide a comprehensive workflow and tutorials, from data preparation to the interpretation of the results, in a dedicated GitHub page https://milesilab.github.io/BioRssay/. Further, the package carries example data sets for self-tests, and more data can be downloaded at https://github.com/milesilab/DATA. The code is maintained by P. Karunarathne (piyalkarumail@yahoo.com). For suggestions or further development please contact the corresponding authors, P. Labbé and P. Milesi.

The *BioRssay* package can be installed in the R environment using the following code: 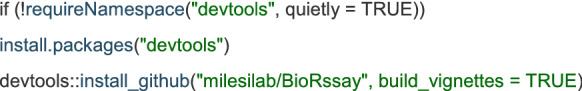


## Data Availability

Accessible at https://github.com/milesilab/DATA; Example 1 and 2 (Fig. 2 and Table 2) can be reproduced using the following dataset included in the package: 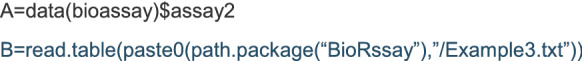

## Abbreviations

LD: Lethal dose
WHO: World Health Organization
CI: Confidence interval
LRT: Likelihood ratio tests
RR: Resistance ratio

## References

[1] Carson R. Silent Spring. Houghton Mifflin Company; 1962.

[2] Labbé P, David J-P, Alout H, Milesi P, Djogbénou L, Pasteur N, et al. 14 - Evolution of Resistance to Insecticide in Disease Vectors. In: Tibayrenc MBT-G and E of ID Second E, editor. London: Elsevier; 2017. p. 313–39

[3] Milesi P, Pocquet N, Labbé P. BioRssay: a R script for bioassay analyses. 2013. https://drive.google.com/file/d/1qMNC2EQlxBnOunuaauta1BCQcLesnrFX/view?usp=sharing

[4] Alout H, Labbé P, Berthomieu A, Makoundou P, Fort P, Pasteur N (2016). High chlorpyrifos resistance in *Culex pipiens* mosquitoes: strong synergy between resistance genes. Heredity (Edinb)..

[5] Pocquet N, Darriet F, Zumbo B, Milesi P, Thiria J, Bernard V (2014). Insecticide resistance in disease vectors from Mayotte: an opportunity for integrated vector management. Parasit Vectors Springer.

[6] Badolo A, Sombié A, Pignatelli PM, Sanon A, Yaméogo F, Wangrawa DW (2019). Insecticide resistance levels and mechanisms in *Aedes aegypti* populations in and around Ouagadougou. Burkina Faso. PLoS Negl Trop Dis..

[7] Assogba BS, Milesi P, Djogbénou LS, Berthomieu A, Makoundou P, Baba-Moussa LS (2016). The ace-1 locus is amplified in all resistant *Anopheles gambiae* mosquitoes: fitness consequences of homogeneous and heterogeneous duplications. PLoS Biol.

[8] Yaméogo F, Wangrawa DW, Sombié A, Sanon A, Badolo A (2021). Insecticidal activity of essential oils from six aromatic plants against *Aedes aegypti*, dengue vector from two localities of Ouagadougou. Burkina Faso. Arthropod Plant Interact..

[9] Epelboin Y, Wang L, Giai Gianetto Q, Choumet V, Gaborit P, Issaly J (2021). CYP450 core involvement in multiple resistance strains of *Aedes aegypti* from French Guiana highlighted by proteomics, molecular and biochemical studies. PLoS ONE.

[10] Perrier S, Moreau E, Deshayes C, El-Adouzi M, Goven D, Chandre F (2021). Compensatory mechanisms in resistant *Anopheles gambiae* AcerKis and KdrKis neurons modulate insecticide-based mosquito control. Commun Biol.

[11] R Core Team. A language and environment for statistical computing, R Foundation for Statistical Computing; 2013. 2020.

[12] Abbott WS (1925). A method of computing the effectiveness of an insecticide. J Econ Entomol College Park.

[13] Johnson RM, Dahlgren L, Siegfried BD, Ellis MD (2013). Acaricide, fungicide and drug interactions in honey bees (Apis mellifera). PLoS ONE.

[14] Finney DJ (1971). Probit analysis.

[15] Robertson JL, Preisler HK (1992). Pesticide bioassays with arthropods.

[16] Holm S (1979). A simple sequentially rejective multiple test procedure. Scand J Stat.

[17] Ritz C, Baty F, Streibig JC, Gerhard D (2015). Dose-response analysis using R. PLoS ONE.

[18] Quast J-P, Schuster D. Protti: Bottom-up Proteomics and LiP-MS Quality Control and Data Analysis Tools. 2021.

[19] Holst KK, Budtz-Jørgensen E. Linear latent variable models: The lava-package. Comput Stat. 2013.

